# 前纵隔巨大肿物1例

**DOI:** 10.3779/j.issn.1009-3419.2012.05.12

**Published:** 2012-05-20

**Authors:** 瑞丽 韩, 殿胜 钟, 小丽 王

**Affiliations:** 300052 天津，天津医科大学总医院呼吸科 Department of Respiratory Medicine, Tianjin Medical University General Hospital, Tianjin 300052, China

## 1 临床资料

患者，女性，24岁，主因“间断咳嗽、咳痰10月，发现纵隔肿物2天”于2011年10月26日入院。患者10个月来间断出现咳嗽，与闻刺激性气体无关，伴少量咳痰，每次持续约1周，无发烧、胸痛、胸闷和气促。自述6个月前曾在外院查胸部X线，未见异常。入院前2天在我院查胸部CT：“前纵隔不规则软组织密度影，最大径线约为57 mm，其内密度不均，病变与升主动脉及肺动脉分界不清，邻近肺组织受压”。为进一步诊治收住我院。患者自发病以来，精神、食欲可，体重无著变，运动正常，四肢有力。既往否认结核病史；否认放射性物质和化学物接触史。

入院查体：发育正常，营养中等，全身浅表淋巴结未触及；气管居中，胸廓对称，双侧呼吸动度一致，叩诊清音，双肺呼吸音清，未闻及干湿啰音；心率72次/分，律齐，各瓣膜听诊区未闻及杂音；腹软，肝脾肋下未触及；双下肢不肿，无杵状指/趾。

诊疗过程：入院后查血、尿、便常规、肝肾功能、电解质等均正常，肿瘤标志物（甲胎蛋白、铁蛋白、癌胚抗原、糖链抗原19-9、糖链抗原242、糖链抗原153、神经原稀醇化酶、细胞角蛋白19片段、鳞状细胞癌抗原）正常。胸部增强CT（图 1）：“前纵隔不规则肿块，侵犯左头臂静脉，并突向左上叶，不除外心包受侵，肿块向上达主动脉弓上约3 cm，向下延伸至左心房下部水平，左头臂静脉受压变窄，增强检查后肿块呈明显不均匀强化，其内可见片状无强化坏死区，纵隔内未见肿大淋巴结影。考虑恶性肿瘤性病变，可能为胸腺上皮来源肿瘤、淋巴源性肿瘤或生殖细胞来源肿瘤”。SPECT示：“胸骨柄异常示踪剂浓集区，结合临床，不除外纵隔肿物局部侵犯”。腹部B超、头颅CT和肺功能检查均正常。经我院肺外科会诊，首先考虑侵袭性胸腺瘤或胸腺癌，遂转入肺外科行“前纵隔肿物切除术”，病理回报（图 2）：“前纵隔大B细胞淋巴瘤，侵犯（心包）、（左头臂）静脉管壁全层及（左下）肺组织；免疫组化染色示：癌细胞呈CD20弥漫性阳性，PLAP、CK19A和CD3阴性”。患者术后恢复良好，后转到血液科进一步化疗，目前在随访中。

**1:**
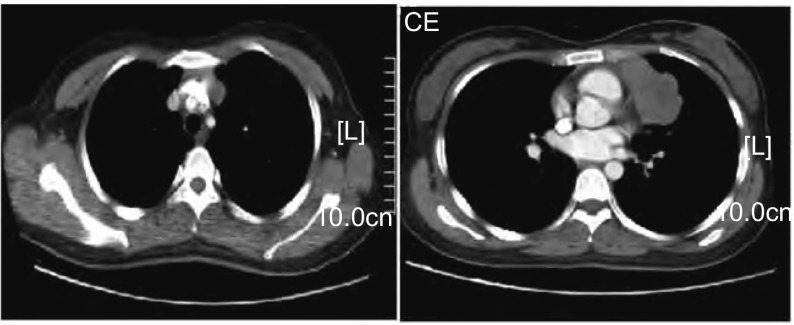
胸部强化CT示：前纵隔不规则肿块，侵犯左头臂静脉，并突向左上叶，增强检查后肿块呈明显不均匀强化，其内可见片状无强化坏死区。 The chest enhancement CT scan revealed an irregular anterior-mediastinal mass with invasion of the left brachiocephalic vein and the left upper lobule of the lung. The bulky lesion was obviously unequal strengthening with contrast-enhanced CT scans, and it was visible flake necrotic area.

**2:**
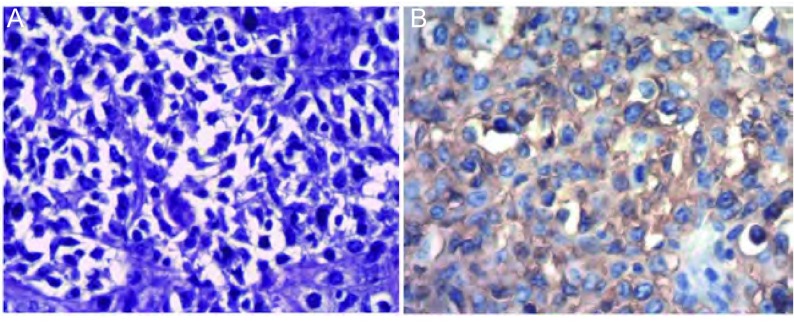
病理结果显示：弥漫增生的大小不等瘤细胞及细胞间不同程度的纤维化（A）（HE, ×400）；免疫组化染色显示：癌细胞呈CD20弥漫性阳性（B）（×400）。 Histological examination demonstrated diffuse hyperplasia of tumor cells with different sizes and extra-cellar matrix fibrosis with different degrees (A) (HE, ×400); B: Immunostaining of the cells demonstrated that neoplastic cells were marked with CD20 diffusely (×400).

## 2 讨论

患者为年轻女性，既往体健，临床特点为迅速增大的前纵隔巨大肿物（< 6个月）伴间断性咳嗽，胸部增强CT显示肿块侵犯左头臂静脉和心包，并呈明显不均匀强化，其内可见片状无强化坏死区，上述临床和影像学表现高度提示患者为前纵隔的恶性肿瘤。临床上常发生于前纵隔的恶性肿瘤包括侵袭性胸腺瘤或胸腺癌、淋巴瘤及恶性畸胎瘤等。胸腺瘤因其独特的解剖位置，是前纵隔最常见的肿瘤，可分为非侵袭性胸腺瘤和侵袭性胸腺瘤或胸腺癌。有趣的是，表现为重症肌无力的胸腺瘤通常较小且常非侵犯性，而侵袭性胸腺瘤或胸腺癌则很少合并重症肌无力。纵隔淋巴瘤临床上以无痛性纵隔淋巴结肿大为典型表现，多与颈部或全身淋巴结肿大同时出现，亦可首先发生于纵隔，但比较少见。本例患者，仔细追问病史，无重症肌无力表现，无全身浅表淋巴结肿大，胸部CT显示纵隔内未见肿大淋巴结影，此外，肿块内可见片状无强化坏死区，但无骨、牙齿等组织的异常钙化影，综上所述，首先考虑为侵袭性胸腺瘤或胸腺癌，淋巴瘤待除外，而恶性畸胎瘤的可能性比较小。需要指出的是，前纵隔肿物的临床和影像学表现缺乏特异性，组织病理学诊断是金标准。标本的获取包括经皮穿刺组织活检术、纵隔镜和外科手术探查。最初，拟行CT引导下经皮纵隔肿块活检，但是，一方面由于患者家属不愿意接受，另一方面临床上高度怀疑为侵袭性胸腺瘤或胸腺癌，而该病首选手术切除，因此转入肺外科，行“前纵隔肿物切除术”，术后病理回报为原发性纵隔大B细胞淋巴瘤（primary mediastinal large B-cell lymphoma, PMBCL）。

PMBCL，起源于胸腺髓样B细胞，故常发生于前纵隔。1980年由Lichtenstein等^[1]^首次报道，1994年欧亚淋巴瘤分类及1999年WHO分类将其定义为弥漫性大B细胞淋巴瘤（diffuse large B-cell lymphoma, DLBCL）的一个亚型^[2]^，占所有非霍奇金淋巴瘤的2%，占DLBCL的6%-10%^[3]^；好发年龄为30岁-40岁，男女发病率之比为1:2。PMBCL生长速度较快，早期多无临床症状，后常因肿物迅速增大挤压和侵袭周围组织引起临床症状而就诊，常见的临床表现包括咳嗽、胸闷、胸痛、呼吸困难及上腔静脉受压的征象等。由于该肿瘤为淋巴结外病变，故初诊患者全身淋巴结受累的很少见，累及骨髓者极为罕见。本患者属于好发年龄，前纵隔肿物生长迅速，且无全身浅表淋巴结及纵隔淋巴结的肿大。

PMBCL肿物直径多大于10 cm（60%-70%），可挤压和侵袭周围组织，如肺、胸壁和心包等，合并坏死时可见低密度影，无异常钙化影。本患者的胸部CT表现符合上述特点，但也必须指出，PMBCL的影像学表现缺乏特异性，需与好发于前纵隔的其它恶性肿瘤进行鉴别，如侵袭性胸腺瘤或胸腺癌、恶性畸胎瘤、其它类型淋巴瘤及纵隔型肺癌等，有时很难鉴别，而最终的确诊往往需要依靠组织病理学检查。CT引导下经皮穿刺组织活检是一个非常简单实用的方法，可获得病理学的诊断，但有时因为取材过小影响诊断的正确性。纵隔镜和开胸活检手术可以获得足够的标本，从而明确诊断，还可以了解纵隔内结构受侵情况，但创伤较大。

PMBCL组织细胞学改变以弥漫增生的大小不等瘤细胞及细胞间不同程度的纤维化为特征，部分瘤细胞胞体较大，胞浆透明，形成所谓的“陷窝细胞”（lacunar cells）；在免疫组化方面，瘤细胞可表达B细胞相关抗原（如CD19、CD20、CD22、C79a等）^[4]^。

目前，关于PMBCL的治疗还没有一个完美的方案，原则上是以化疗为主的综合治疗，因为PMBCL属于DLBCL的一个亚型，化疗方案类同于DLBCL，即CHOP方案（环磷酰胺、阿霉素、长春新碱和强的松）。妥昔单抗（美罗华）可使DLBCL治疗的有效率和总生存率均提高15%-20%，但对于PMBCL的疗效尚需要观察。考虑到放疗对心脏和肺的影响，化疗后是否常规放疗仍存在争议。对于合并上腔静脉综合征等压迫症状且进展迅速，为迅速解除局部压迫症状并同时获取病理结果，可行肿瘤姑息切除术。PMBCL的预后较好，5年总生存率可达50%-80%。

总之，PMBCL是一种具有独特临床特征的DLBCL，表现为发展迅速的前纵隔巨大肿物，并可累及周围的组织及合并坏死，且可无全身浅表淋巴结及纵隔淋巴结的肿大，临床上经常需要与其它好发于前纵隔的恶性肿瘤进行鉴别，组织病理学是确诊的关键，治疗上是以化疗为主的综合治疗，预后较好。由于本病不属于临床常见病，临床医师尤其是呼吸科医师应该提高对本病的认识，从而使患者得到及时准确的诊断和治疗。
